# The Chemokines CXC, CC and C in the Pathogenesis of COVID-19 Disease and as Surrogates of Vaccine-Induced Innate and Adaptive Protective Responses

**DOI:** 10.3390/vaccines10081299

**Published:** 2022-08-11

**Authors:** Mojgan Noroozi Karimabad, Gholamhossein Hassanshahi, Nicholas G. Kounis, Virginia Mplani, Pavlos Roditis, Christos Gogos, Maria Lagadinou, Stelios F. Assimakopoulos, Periklis Dousdampanis, Ioanna Koniari

**Affiliations:** 1 Molecular Medicine Research Center, Research Institute of Basic Medical Sciences, Rafsanjan University of Medical Sciences, Rafsanjan 7717933777, Iran; 2 Department of Internal Medicine, Division of Cardiology, University of Patras Medical School, 26500 Patras, Greece; 3 Intensive Care Unit, Patras University Hospital, 26500 Patras, Greece; 4 Department of Cardiology, Mamatsio Kozanis General Hospital, 50100 Kozani, Greece; 5 COVID-19 Unit, Papageorgiou General Hospital, 56403 Thessaloniki, Greece; 6 Department of Internal Medicine, Division of Infectious Diseases, University of Patras Medical School, 26500 Patras, Greece; 7 Department of Nephrology, Saint Andrews State General Hospital, 26221 Patras, Greece; 8 Department of Cardiology, University Hospital of South Manchester, NHS Foundation Trust, Manchester M23 9LT, UK

**Keywords:** CXC chemokines, COVID-19, coronavirus, CC chemokines, virus

## Abstract

COVID-19 is one of the progressive viral pandemics that originated from East Asia. COVID-19 or SARS-CoV-2 has been shown to be associated with a chain of physio-pathological mechanisms that are basically immunological in nature. In addition, chemokines have been proposed as a subgroup of chemotactic cytokines with different activities ranging from leukocyte recruitment to injury sites, irritation, and inflammation to angiostasis and angiogenesis. Therefore, researchers have categorized the chemotactic elements into four classes, including CX3C, CXC, CC, and C, based on the location of the cysteine motifs in their structures. Considering the severe cases of COVID-19, the hyperproduction of particular chemokines occurring in lung tissue as well as pro-inflammatory cytokines significantly worsen the disease prognosis. According to the studies conducted in the field documenting the changing expression of CXC and CC chemokines in COVID-19 cases, the CC and CXC chemokines contribute to this pandemic, and their impact could reflect the development of reasonable strategies for COVID-19 management. The CC and the CXC families of chemokines are important in host immunity to viral infections and along with other biomarkers can serve as the surrogates of vaccine-induced innate and adaptive protective responses, facilitating the improvement of vaccine efficacy. Furthermore, the immunogenicity elicited by the chemokine response to adenovirus vector vaccines may constitute the basis of vaccine-induced immune thrombotic thrombocytopaenia.

## 1. Introduction

A group of patients suffering from viral pneumonia in Wuhan, China, in December 2019 was diagnosed with inflammation by a new Coronavirus, namely, severe acute respiratory syndrome coronavirus-2 (SARS-CoV-2) [1]. According to medical examinations, SARS-CoV-2 causes a variety of conditions ranging from moderate upper respiratory tract infection, mild infection of the lungs with radiological abnormalities, and asymptomatic infection to severe viral pneumonia with respiratory failure and in some cases death [2]. The epidemiological and scientific characteristics of those suffering from COVID-19 are remarkable; however, the pathogenesis of SARS-CoV-2 infection has not been properly elucidated [3]. Immunohistochemical research has demonstrated the presence of numerous chemokine ligands in parallel with their receptors in COVID-19 lesions [4]. Chemokines, interleukins (IL), interferons (INF), tumor necrosis factors (TNF), and colony-stimulating factors constitute the cytokine network that is a complex and excessive immune response triggered by a variety of external stimuli such as severe viral infection. Consequently, chemokines contribute crucially to COVID-19 pathogenesis [4]. Chemokines (from the Greek χυμεία (khumeíā) and κίνησις (kī́nēsis) ‘movement’) or chemotactic cytokines are a subgroup of small cytokines or signaling proteins secreted by cells that induce the directional movement of leukocytes—as well as other cell types, including endothelial and epithelial cells—to specific sites within tissues during various inflammatory conditions [5]. Researchers have identified chemokines as a subfamily of the larger family of cytokines, which are used as the recruiter/migratory elements for various cells, and their target cells have specific transmembrane G-protein chemokine receptors. Moreover, chemokines have been subdivided into four sub-groups consistent with the position of the cysteine motifs of their structure. These sub-groups include CXC (called α-chemokines), CC (called β-chemokines), C that have only two cysteines (called γ chemokines), andCX3Cthat have three amino acids between the two cysteines (called d-chemokines). The aberrant and immoderate production of cytokines has been linked to morbidity and mortality in several viral infections [5]. Additionally, it has been found that the hyper-induction of pro-inflammatory cytokines plays a vital role in the development of infections with SARS-CoV [6], SARS-CoV-2 [7], and Middle East respiratory Syndrome coronavirus (MERS-CoV) [7]. Studies have found that pneumonia is the most important concern after a SARS-CoV-2 infection, which manifests as acute respiratory distress syndrome (ARDS) [2]. The first coordinated protective defense line of the body against tissue damage and infection has been proposed to be inflammation, because it activates both the innate and adaptive immune responses [8]. However, intensive immune responses due to infection, also termed a cytokine storm, have been shown to be related to immoderate ranges of pro-inflammatory cytokines and tissue damage [9]. Moreover, a cytokine storm is a condition observed in the course of infection with influenza virus [10] as well as with coronaviruses [11], which contributes to acute lung damage and the development of ARDS. In this regard, studies have proven that cytokine storms and the outcomes of various cytokines’ induction are stimulated by SARS-CoV-2 infection [7]. However, the identity of the biomarkers responsible for the disorder’s severity and progression should be further clarified. Researchers have also identified more than 20 chemokine receptors and 50 chemokines [12]. Several members of the CXC chemokine family have been shown to be implicated in inflammatory conditions, including atopic dermatitis, alopecia areata, Behcet’s syndrome, diabetes mellitus, various infections, diabetes [13,14], multiple sclerosis [15], food allergies [16], pre-eclampsia [17], osteoporosis [18], hepatitis B virus (HBV) [19], and systemic lupus erythematosus (SLE). The physiopathology of the COVID-19 pandemic and specific chemokines contribute significantly to this condition. However, a clear and linear relationship between chemokines and COVID-19 has not been fully elucidated. The objective of this study was to review the physiopathology of the COVID-19 pandemic and certain chemokines especially from the CXC and CC sub-groups that seem to play a key role in this regard. By recruiting innate and adaptive immune cells to areas of infection, enhancing their cytotoxic function, and producing antiviral mediators, the chemokines play an important role in fighting viral infections. However, some large DNA viruses (e.g., herpesviruses) have the ability to mimic the chemokines and escape the immune system, a fact that leads to viral propagation and disease persistence [20,21]. The goal of this study is to review information on the chemokines influencing COVID-19 progression and associated vaccination. The analysis of each chemokine and its specific role during the COVID-19 infection will help with the elucidation of the development of complications and further prevention by identifying the possible outcome of the disease and any available targeted therapies against chemokines or their receptors in the ongoing related clinical trials [21].

## 2. Methods

We conducted a review of the literature on PubMed, Medline, and Embase databases via Google using the terms ‘chemokines’, ‘CC chemokines’, ‘CXC chemokines’, and ‘COVID-19’ as “Title/Abstract” or as “MeSH Terms”. The structure of the search in the “Search details” window of the PubMed website was chemokines, COVID-19 pathogenesis, and vaccine induced response. We also reviewed the references of all identified manuscripts to identify additional relevant publications. All observational studies and randomized trials on the role of chemokines during the COVID-19 infection were included. Case reports, case series, and studies not including adults were excluded. Only publications in English were included.

## 3. Bio-Structure and Functions of Chemokines

As mentioned earlier, chemokines have been introduced as a family of relatively small, highly conserved proteins (8 to 12 kDa) that are involved in many biological functions, including chemotaxis [22], leukocyte degranulation [23], hematopoiesis [24], and finally angiogenesis [25]. Researchers have normally classified chemokines into subfamilies based on the sequential position of the first two of their four cysteine residues, namely CC and CX3C or CXC [26]. One exception is the C chemokine subfamily due to its disposal of just one N-terminal cysteine residue. However, in the largest sub-families, namely, CXC and CC, the first cysteines are close to the CC motif or separated by one amino acid residue (CXC motif). The C type chemokines do not have the first and 1/3 of those cysteines but the CX3C chemokines possess three amino acids between the primary cysteine residues. Although sequence homology between chemokines varies between approximately 20% and 90%, their universal sequences have been identified to be quite conserved [27].

## 4. Chemokines and Pathologic Disorders

It is widely accepted that chemokines have a major function in biology and are involved in many pathologic disorders such as cancer, atherosclerosis, and HIV/AIDS [28]. Nearly 50 chemokines have been considered to play a role in immunological processes. In general, chemokines stimulate their targets by binding to cellular G-protein coupled receptors (GPCRs) [29]. Researchers have shown that proangiogenic effects have been attributed toCXCL12/stromal cell-derived factor-1 (SDF-1) that alternatively binds to CXCR4 and ACKR3/CXCR7 receptors [30]. However, a good way for understanding how chemokines act on receptors has been shown by structural information of the chemokine ligand/receptor complex [31].

## 5. Chemokines and ARDS

From the first reviews on COVID-19, analyses have shown pneumonia as the most frequent medical manifestation. COVID-19-related pneumonia may be subsequently accompanied with ARDS, which is currently considered a hallmark of the immune-mediated clinical effect in SARS-CoV-2, and is consistent with the extremely acute respiratory syndrome (SARS-CoV) and MERS-CoV infections as two different varieties of acute respiratory infections resulting from Coronaviruses [32]. Moreover, ARDS can be described as a generalized and uncontrolled inflammatory response, which is potentially responsible for a wide range of morbidities and mortalities amongst the infected cases. In addition, ARDS may arise in a variety of conditions such as sepsis, pneumonia, blood transfusion, and pancreatitis related to an increased mortality [33]. A cytokine storm takes place through infection with numerous viruses, such as influenza viruses [2] and Coronaviruses [34], and has been proposed as a factor involved in acute lung damage and the development of ARDS. Furthermore, during COVID-19 development, ARDS is the main critical outcome of the so-called “cytokine storm” [35]. The study of Ichikawa et al. (2012) demonstrated the activation of chemokines such asCXCL10-CXCR3-signaling in ARDS, which worsened the effect in mice [36].

## 6. Chemokines in Viral Diseases

Immune cells are recruited by chemokines to the infection site to combat the intruder [37]. Results have indicated the association of viral infections with the expression of numerous chemokines, particularly the interferon-inducible ones. Interferons (IFN) that may be induced with different cellular mechanisms can be implicated in fast and effective host innate responses against viruses. An effective IFN reaction stimulated through primary contact with a virus could decelerate viral multiplication and “buy time” for the respective organism to establish more effective adaptive immune reactions [38]. In fact, IFNs are capable of stimulating the surrounding cells for expressing potent anti-viral proteins together with enzymes, transcription elements, cell surface glycoproteins, chemokines, and cytokines [39]. In addition, IFN are capable of inhibiting cellular proliferation or rapid growth, as well as further modifying apoptosis and modulation. Coperchini et al. have reviewed 53 (2020) immune reactions associated with cytokine activation [38,39,40]. Additionally, the chemokines contribute to immune cell trafficking and inflammation during viral infections. Chemokines perform a main role in preliminary management of the respiratory tract infections because of their chemotactic activities towards monocytes and neutrophils [41]. Chemokine production within nasal washing fluids have a correlation with the severity of acute infections in the respiratory tract [42]. Despite the fact that most people with manifest chemokine activation could correctly deal with viral infections, several viruses can escape from such a surveillance system. Furthermore, viruses can use the chemokine network for their own function using several strategies. Some viruses “mimic” the features of chemokines via generating molecules, which may be similar to chemokines and may interact with their receptors. These molecules play a crucial role in controlling the immune response [43]. SARS-CoV employs multiple passive and active mechanisms to avoid the induction of the antiviral type I interferons in tissue cells [44]. All of the above denote that viruses have the ability to interfere with the chemokine and/or chemokine-receptors as they can modify the intracellular signaling further causing the dissemination of infection. A hallmark of SARS is the systemic infection and cytokine storm with higher levels of IL-8, IL-6, CXCL10, CCL2, and CCL3 [45].

## 7. Chemokines and COVID-19

The function of chemokines as robust chemo-attractants involves recruiting the inflammatory cells to emigrate from intravascular areas across the epithelium and endothelium into the inflammation sites maintaining a chemokine gradient [46]. The instantaneous immune responses to infection by bacteria, viruses, or other microorganisms involves mobilizing the molecules and cells as well as activating enzymatic, biosynthetic, energetic, and metabolic sources [47,48]. In fact, the metabolic dysfunction as a result of viral contamination is responsible for re-programing the host metabolism to generate powerful anti-viral protection responses. The data on the interferences between the viral function and chemokines have revealed the molecular mechanisms underlying the adaptive immune response’s reaction to viral infection [48] regarding COVID-19, which defined a distinct inflammatory reaction related to that of SARS-CoV-2 infection. Research has revealed that a “beside the point and weak immune reaction” emerges frequently in sufferers with comorbidities. Therefore, such a condition may cause virus replication leading to disease complications [49]. It is notable that inflammatory responses in viral pneumonia have been considered a double-edged sword. Although inflammation is essential for infection control, the exaggerated inflammatory responses in individuals with pneumonia can further lead to the excessive release of inflammatory cytokines called a “cytokine storm”, resulting in unfavorable effects including progressive respiration failure, the diffusion of alveolar damage and fibrosis, and multiple organ failure. There have been reviews and published records on the changing expression of CXC and CC chemokines in patients with COVID-19. A Summary of the literature review in relation to immune-related diseases is reported in Table 1.

### 7.1. CXC Chemokines and COVID-19

#### 7.1.1. CXCL1

According to Nima Hemmat et al.’s hypothesis, neutrophils are essential immune cells involved in SARS-CoV infection that enhance hemorrhagic lesions and infection within the lungs of affected patients. ARG1, CXCL1, and ELANE promote neutrophilia in the infected cases through their over expression. For this reason, inhibitors of Serpins and Arginase at some stage of SARS-CoV infection may contribute to the survival of those infected with SARS. Considering the higher similarities of SARS-CoV-2 to SARS-CoV, the application of the above inhibitors is probably preferred for COVID-19 sufferers [50]. Abby et al. have determined that the genes implicated in inflammation, including CXCL1 and domain-containing protein 2 (NOD2), are up-regulated in people who smoke and smoke electronic cigarettes. In severe COVID-19 cases, the hallmark of this infection is the release of inflammatory products, which includes IL-1B along with the cytokine storm. Consequently, their findings established that smoking or vaping might significantly exacerbate COVID-19-associated infection or increase susceptibility to COVID-19 [51].

#### 7.1.2. CXCL2

As stated by Islam et al., the analysis of COVID-19 nasopharyngeal specimens and comparative analyses with other SARS-CoV-2 infection models demonstrated different host responses against SARS-CoV-2. Notably, the induction of phagosome, apoptosis, the hypoxia response, as well as antigen presentation were deficient within those cases. The upregulation of the immune and cytokine signaling genes such as CXCL2 has been demonstrated in the lungs [52]. In the study of Miyazawa on SARS-CoV-2, a dangerous cycle of CCL2- and CXCL2-mediated inflammatory monocytes, neutrophil infiltration, and the activation of other molecules along with the tumor necrosis factor (TNF)-α, the TNF-related apoptosis-inducing ligand, and nitric oxide have been shown to be involved in the pathogenesis of tissue damage [53].

#### 7.1.3. CXCL3

Loganathan et al. adopted a host transcriptome-based drug repurposing strategy based on the available gene expression data on SARS-CoV-2 and other respiratory infection viruses. The early infection models of SARS-CoV-2 demonstrated upregulated pro-viral factors such as TYMP, PTGS2, C1S, CFB, IFI44, XAF1, CXCL2, and CXCL3. The direct inhibition of the PTGS2 gene product can be regarded as a therapy target for SARS-CoV-2 infection [54].

#### 7.1.4. CXCL4

The smaller chemokine proteins released by activated platelets are platelet factor four (PF4) and CXCL4.Whereas the main physiological feature of PF4 is in blood coagulation, this cytokine is also involved in the adaptive and innate immunity. Zheng et al. showed that a number of approaches for the control of COVID-19 patients might also encompass renal replacement therapy (CRRT) and extra-corporeal membrane oxygenation (ECMO), which additionally require heparin. Moreover, anti-PF4 antibodies have been detected in those with severe COVID-19 as well as heparin-induced thrombocytopenia (HIT). Finally, PF4, its contribution to HIT, and to pathologies in cases suffering from COVID-19 reflect a therapeutic alternative to using blocking antibodies for COVID-19 treatment [55].

#### 7.1.5. CXCL8

According to studies, CXCL8, which is also called IL-8, has been considered together with some other chemokines to be used as a prognostic biomarker for the ARDS course [56]. Certainly, researchers designated CXCL8 for multiplication in plasma [57] and within the bronchoalveolar lavage fluid [58] of ARDS sufferers. One of the direct functions of CXCL8 in ARDS development was demonstrated in rabbits affected with ARDS and resulted in intern-fold augmentation of the CXCL8 levels in alveolar fluids. However, the pre-treatment with an anti-CXCL8 antibody revealed no improvement of standard acute lung injuries [59]. Park et al. indicated that epithelial damage was related to neutrophil infiltration. Moreover, the myeloid cells of acute sufferers confirmed the greater expression of pro-inflammatory cytokines and chemokines along with CXCL8. It has been found that neutrophils are increased in the lungs of cases with greater chemokine expression. Moreover, the detection of genes associated with neutrophil extracellular traps has been observed in the recruited neutrophils. In addition, neutrophil-mediated infection changed into regulation with the aid of glucocorticoid receptor expression and activities. The above outcomes suggested the presence of excessive COVID-19 symptoms through differential expression of neutrophil and glucocorticoid receptors [60].

#### 7.1.6. CXCL9

In a study by Abers et al., a multivariable analysis of samples from 175 Italian patients detected several biomarkers such as CXCL9 that have been substantially related to mortality while the patients’ condition started to improve; indeed, IL-1α is related to mortalities when reduced. Out of those biomarkers, the levels of sTNFRSF1A, sST2, IL-15, and IL-10 were continuously increased in hospitalized patients who died compared to the recovered ones, revealing that the abovementioned biomarkers can also be indicative of an adverse disease outcome/mortality [61]. In the study by Tincati et al., COVID-19 and 20 mild COVID-19 cases were investigated. Severe COVID-19 sufferers demonstrated increased levels of non-classical monocytes, several plasma chemokines such as CXCL9 cytokines (IL-6 and IL-10), and ROS as opposed to mild COVID-19. Furthermore, COVID-19 revealed improved granzyme-B^+^/perforin^+^-induced cytolytic T-cells and activated CD38^+^HLA-DR^+^ T-cells. Finally, a SARS-CoV-2 specific T-cell reaction with a prevalence of Th1 bifunctional or trifunctional IFN-γ/IL-2/TNF-α-expressing CD4^+^ was reported at the same time, while no difference consistent with the disease severity was discovered [62].

#### 7.1.7. CXCL10

A considerable upregulation of CXCL10 expression was observed in the lung following ARDS induction with lipopolysaccharide (LPS) in a mouse model of lung damage; additionally, CXCL10 neutralization was reported with an anti-CXCL10 antibody that resulted in amelioration of lung damage [63]. Cheemarla et al. confirmed that in nasopharyngeal swabs, CXCL10 is increased for the duration of SARS-CoV-2 infection and used a CXCL10- based screening method to identify four undiagnosed cases of COVID-19. They showed that NP CXCL10 had a good overall performance and their outcomes show how biomarker-based screening can be used to leverage the existing PCR testing potential to rapidly permit the large scale testing for COVID-19 [64]. Runfeng et al. confirmed that Lianhua Qingwen drastically inhibited novel SARS-CoV-2 replication in Vero E6 cells, which significantly decreased numerous inflammatory cytokines such as TNF-α, CXCL-10/IP-10, and IL-6 at mRNA level, which could be a new promising method to control COVID-19 [65].

### 7.2. CC Chemokines and COVID-19

#### 7.2.1. CCL2

Kempuraj et al. showed that COVID-19 pathogenesis may be due to a cytokine storm with higher rates of IL-1β, interleukin-6 (IL-6), chemokine (C-C-motif) ligand 2 (CCL2), granulocyte-macrophage colony-stimulating factor (GM-CSF), and tumor necrosis factor-alpha (TNF-α). Their results indicated the ability of COVID-19 to cause neurological disorders and severe pneumonia, including stroke, blood–brain barrier (BBB) disruption, damage to the neuro-vascular structure, higher intra-cranial pro-inflammatory cytokines, and endothelial injury in patients’ brains [66]. In the research by Ray et al., the authors discussed the likelihood of an interaction, which can be a vital factor of the disease severity in COVID-19. A number of the above agents, including CCL2 and epiregulin acting through EGFR receptor and TNFα, may be inhibited by treatments practiced in the past (e.g., CCL2) or with existing therapeutics (e.g., TNFα or EGFR) [67]. Ruan et al. established that the usage of a traditional Chinese prescription called Dayuanyin (DYY) for COVID-19, which acted on key targets consisting ofIL-6, IL-1β1B, and CCL2 via IL-17 and the AGE-RAGE signaling pathways in diabetic complications, as well as cytokine-cytokine receptor interplay. DYY may play an important position in treating COVID-19 via suppressing the inflammatory cycle and regulating immune features [68].

#### 7.2.2. CCL3, CCL4

Gruber et al. have investigated the immune profiles of eight cases of Multisystem Inflammatory Syndrome in children (MIS-C). Each MIS-C patient revealed proof of earlier SARS-CoV-2, mounting an antibody response with daily isotypes with switching and neutralization capabilities. They further profiled the humoral immune response using high-dimensional cytokine assays that demonstrated improved signatures of IL-18 and IL-6, lymphocytic and myeloid chemotaxis, and the activation of CCL3, CCL4, and CDCP1, as well as the immune dysregulation of IL-17A [69]. Xiong et al. highlighted the relationship of COVID-19 pathogenesis to an excess cytokine release consisting of CCL4/MIP1B and CCL3/MIP-1A. In addition, SARS-CoV-2 resulted in an activation of apoptosis and the P53 signaling pathway in lymphocytes, which could be a reason for lymphopenia in these cases. Moreover, the transcriptome dataset of COVID-19 sufferers could be a precious source for scientific research on anti-inflammatory drug development and clarification of molecular mechanisms related to the host response [70]. According to Trump S et al., in COVID-19, hypertension as well as cardio-vascular disease were the main risk factors for the development of severe disease. Angiotensin converting enzyme inhibitor (ACEI) therapy was related to decreased COVID-19-associated hyper-inflammation and an extended innate anti-viral response, while angiotensin receptor blocker (ARB) agent was associated with more desirable epithelial–immune interactions. Finally, the neutrophils and macrophages of sufferers of high blood pressure, especially those under ARB treatment, have shown greater expressions of chemokine receptor CCR1 and inflammatory cytokines such as CCL3 and CCL4 [71].

#### 7.2.3. CCL5

Bruce et al. reported a considerable increase of CCL5 (RANTES) and plasma IL-6, decreased CD8^+^ T-cell levels, and SARS-CoV-2 plasma viremia in 10 terminally-ill, critical COVID-19 cases. Post treatment with CCR5-blocking antibody leronlimab, researchers have shown the CCR5 receptor occupancy on T-cells and macrophages, the restoration of the CD4/CD8 ratio, a tremendous reduction in SARS-CoV-2 plasma viremia, and a fast reduction in plasma IL-6. These results display a unique method for eliminating unchecked inflammation, the restoration of the immunologic deficiency, and the reduction in the SARS-CoV-2 plasma viral load through disrupting the CCL5–CCR5 axis [72]. According to Takahashi et al., who focused their evaluation on individuals with mild to moderate disease not taking immunomodulatory medicines, the male patients showed greater plasma levels of chemokines and innate immune cytokines such as CCL5 and IL-18, alongside an enhanced induction of non-classical monocytes. On the contrary, the female sufferers demonstrated much greater T-cell activation in comparison to the male cases over the course of the SARS-CoV-2 infection, which was more substantial in older age groups [73].

#### 7.2.4. CCL0

Yao and et al. have confirmed that the genes encoding interleukins (IL-1β,IL-1α, and IL-10 and IL-6), chemokines and interferons (IFN-β1, IFN-α2, and IFN2),and chemokine CCL10 increased considerably in COVID-19 patients, which was consistent with the extensive infiltration of the T-cells, monocytes, and natural killer cells in a SARS-CoV-2-treated group at 24 h [74]. Another patient with multiple sclerosis infected with SARS-CoV-2 in the course of fingolimod (immunomodulating medication) therapy that was admitted to the hospital with a mild clinical picture and who recovered within 15 days has been described by Chiarini et al. [75]. Excessive levels of CCL5 and CCL10 chemokines and antibody-secreting B-cells were detected in these patients [75].

#### 7.2.5. CCL17

In the study by Sugiyama, numerous factors such as CCL17 have been recognized as predictors of the initiation of severe/critical symptoms during COVID-19 infection. CCL17 showed a full differentiation between slight/mild and extreme/critical cases at the initial stages of SARS-CoV-2 infection. A lower expression of CCL17 was particularly pronounced in severe COVID-19 sufferers, while the predictive capacity of CCL17 was indicated in confirmed cases of COVID-19. These elements could be attractive prognostic markers for distinguishing the mild/moderate sufferers from the severe ones, which enables triage at the initial stage of infections [76].

#### 7.2.6. CCL19

Balnis et al. showed that CCL19 can potentially simplify the early detection of COVID-19-ARDS patients with a greater mortality risk and that it might constitute one of the new targets for immunotherapy in this setting. They determined the expression levels of 44 circulating cytokines/chemokines in COVID19-ARDS patients and observed that 13 of them correlated with poorer outcomes. Indeed, chemokine CCL19 has been shown to be substantially related to the adverse outcomes (*p* = 0.009). This chemokine was already determined to be greater in an animal version of SARS-CoV-2. In addition, CCL19 is related to the bronchus-associated lymphoid tissue (BALT) preservation and is responsible for lung immunity to influenza virus [77].

#### 7.2.7. CCL20

Epithelial cells in COVID-19 patients were demonstrated to have an average three-fold increase in their expression of SARS-CoV-2 receptor ACE2, which can be correlated with interferon indicators in the immune cells. Compared to moderate instances, critical cases have shown more robust interactions among the immune and epithelial cells, which has been reflected based on ligand-receptor expression profiles as well as the activated immune cells such as inflammatory macrophages that express CCL20 and several chemokines. It should be noted that transcriptional variations in critical cases in comparison to mild ones can reflect the greater inflammatory damage to the tissue, respiratory failure, and lung damage [78]. Immunity against SARS-CoV-2 would be obtained using convalescent COVID-19 sufferers in connection with (A) a Th17 cellular apparatus in the psoriatic dermis and (B) a lately determined phenomenon wherein Th17 cells would be transformed into the tissue-resident reminiscence T cells with the Th1 phenotype. In addition, during infection, neutrophils secrete IL-17A that can induce the lung epithelial cells toward specific CCL20 production. Natural Th17cells have been also been demonstrated in the infection site using CCL20 and are further increased in the presence of IL-23 [79].

**Table 1:** Summary of the Literature Reviewed in Relation to the Immune-Related Diseases.

First Author	Country	Other Name	Chemokine Receptors	Technique Employed	Chemokine	Presentation on Immune Cells	Role in Immunity	Kind of Chemokine	Expression and Role in COVID-19	Reference
Hemmat N, et al.	Iran	(GRO-a)	CXCR2	Clinical trial	CXCL1	Mesenchymal stem cells (MSC), neutrophil, monocyte	Migration of neutrophils	CXC chemokine	CXCL1 enhance the neutrophilia condition in these patients by their overexpression	[48]
Lee AC, et al.	USA	(GRO-a)	CXCR2	Prospective Clinical trial	CXCL1	MSC, neutrophil, monocyte	Migration of neutrophils	CXC chemokine	Smoking or vaping, by the dysregulation of key genes such as CXCL1, critically exacerbate COVID-19-related inflammation	[49]
Islam A, et al.	Bangladesh	(GRO-b, MIP-2a)	CXCR2	Retrospective clinical trial	CXCL2	MSC, neutrophil, monocyte	Migration of neutrophils	CXC chemokine	Upregulation of the immune and cytokine signaling genes consisting of CXCL2 were determined in lungs	[50]
Miyazawa M, et al.	Japan	(GRO-b, MIP-2a)	CXCR2	Prospective clinical trial	CXCL2	MSC, neutrophil, monocyte	Migration of neutrophils	CXC chemokine	A dangerous cycle of CCL2- and CXCL2-mediated inflammatory monocyte- and neutrophil-related apoptosis	[51]
Loganathan T, et al.	India	GRO3	CXCR2	Prospective clinical trial	CXCL3	MSC, neutrophil, monocyte		CXC chemokine	The up-regulated CXCL3 were recognized in early infection models of SARS-CoV-2.	[52]
Cai Z, et al.	USA	PF4	CXCR3	Review	CXCL4	neutrophil, monocyte		CXC chemokine	CXCL4 can be a therapeutic alternative to the use of blocking antibodies within the COVID-19 remedies	[53]
Park JH, et al.	South Korea	(IL-8)	CXCR1 CXCR2	Prospective clinical trial	CXCL8	MSC, neutrophil, monocyte	Migration of neutrophils	CXC chemokine	In COVID-19, confirmed greater expression of pro-inflammatory cytokines and chemokines along with CXCL8.	[58]
Abers MS, et al.	USA	(MIG)	CXCR3-A/B	Prospective clinical trial	CXCL9	MSC, T cell, microvascular cells	Migration of Th1, CD8 and NK	CXC chemokine	Discovered several biomarkers such as CXCL9 that have been substantially related to mortality	[59]
Tincati C, et al.	Italy	(IP-10)	CXCR3-A/B	Meta-analysis	CXCL9	MSC, T cell, microvascular cells	Migration of Th1, CD8 and NK cells	CXC chemokine	COVID-19 patients displayed higher non-classical monocytes, plasma chemokines CXCL8, CXCL9, CXCL10	[60]
Cheemarla NR, et al.	USA New Haven	(IP-10)	CXCR3-A/B	Prospective clinical trial	CXCL10	MSC, T cell, microvascular cells	Th1 response Migration of Th1, CD8 and NK cells	CXC chemokine	CXCL10 is increased for the duration of SARS-CoV-2 infection	[62]
Runfeng L, et al.	China	(IP-10)	CXCR3-A/B	Prospective clinical trial	CXCL10	MSC, T cell, microvascular cells	Th1 response Migration of Th1, CD8 and NK cells	CXC chemokine	The drug Lianhuaqingwen (LH)significantly decreased numerous seasoned-inflammatory cytokines as such CXCL-10/IP-10 manufacturing on the mRNA ranges	[63]
Kempuraj D, et al.	USA	MCP-1	CCR2	Preprint study	CCL2	MSC, monocyte, T cell, DC	Migration of inflammatory monocytes	CC chemokine	The hallmark of COVID-19 pathogenesis is with elevated levels of CCL2	[64]
Ray PR, et al.	USA	MCP-1	CCR2	Prospective clinical trial	CCL2	MSC, monocyte, T cell, DC	Migration of inflammatory monocytes	CC chemokine	CCL2 inhibitor drugs for treating high risk or severe COVID-19 cases	[65]
Ruan X, et al.		MCP-1	CCR2	Review	CCL2	MSC, monocyte, T cell, DC	Migration of inflammatory monocytes	CC chemokine	Dayuanyin (DYY) treatment of COVID-19 via suppressing the inflammatory typhoon such as CCL2 and regulating immune characteristics	[66]
Gruber C et al.	USA	(MIP-1a) (MIP-1b)	CCR1, CCR5	Prospective clinical trial	CCL3, CCL4	MSC, monocyte, T cell, DC, HSC	Migration of macrophages and NK cells T cell/DCs interaction Migration of macrophages and NK cells T cell/DCs interaction	CC chemokine	Cytokine profiling identified elevated signatures of lymphocytic and myeloid chemotaxis and activation of CCL3, CCL4, and CDCP1	[67]
Xiong Y, et al.	China	(MIP-1a) (MIP-1b)	CCR1, CCR5	Prospective clinical trial	CCL3, CCL4	MSC, monocyte, T cell, DC, HSC	Migration of macrophages and NK cells T cell/DCs interaction Migration of macrophages and NK cellsT cell/DCs interaction	CC chemokine	Excess cytokine release consisting of CCL4/MIP1B and CCL3/MIP-1A In SARS-CoV-2	[68]
Trump S	Germany	(MIP-1a) (MIP-1b)	CCR1, CCR5	Clinical trial (preprint)	CCL3, CCL4	MSC, monocyte, T cell, DC, HSC	Migration of macrophages and NK cells T cell/DCs interaction Migration of macrophages and NK cells T cell/DCs interaction	CC chemokine	Exhibited higher expression of the pro-inflammatory cytokines CCL3 and CCL4 and the chemokine receptor CCR1	[69]
Patterson BK	USA	(RANTES)	CCR1, CCR3, CCR5	Prospective clinical trial	CCL5	MSC, T cell, DC	Migration of macrophages and NK cells	CC chemokine	Reported profound elevation of plasma IL-6 and CCL5 (RANTES), decreased CD8^+^ T cell levels, and SARS-CoV-2 plasma viremia.	[70]
Takahashi T, et al.	USA	(RANTES)	CCR1, CCR3, CCR5	Clinical trial	CCL5	MSC, T cell, DC	Migration of macrophages and NK cells	CC chemokine	CCL5 increased	[71]
Yao Z, et al.	China	(MIP-1 gamma), also called MIP-2	CCR1	Prospective clinical trial	CCL10	monocytes and NK cells	Migration of macrophages and NK cells	CC chemokine	Enhancement of CCL10	[72]
Marco Chiarin, M, et al.	Italy	MIP	CCR1	Prospective clinical trial	CCL10	monocytes and NK cells	Migration of macrophages and NK cells	CC chemokine	Excessive tiers of CCL5 and CCL10 chemokines were detected	[73]
Sugiyama M, et al.	Japan	TARC	CCR4	Prospective clinical trial	CCL17	MSC, T cell, macrophage, DC	T cell/DCs interaction Migration of monocytes	CC chemokine	Upregulated early post SARS-CoV-2 infection	[74]
Balnis J, et al.	Albany	(MIP-3b)	CCR7	Case report	CCL19	T cell, macrophage, DC	T cell and DC homing to lymph node	CC chemokine	Higher plasma levels of Chemokine CCL19	[75]
Chua RL	Germany	(MIP-3a)	CCR6	Comprehensive analysis	CCL20	macrophage Neutrophils	Th17 responses	CC chemokine	Compared to moderate cases, critical cases exhibited stronger interactions between epithelial and immune cells, including inflammatory macrophages expressing CCL20.	[76]
Katayama H, et al.	Japan	(MIP-3a)	CCR6	Clinical trial	CCL20	macrophage Neutrophils epithelial cells	Th17 responses	CC chemokine	Stimulates lung epithelial cells to express CCL20 and increased CCL20	[77]
Jain R, et al.	Dubai		CCR4	Clinical trial	CCL22	MSC, T cell, macrophage, DC	T cell/DCs interaction Migration of macrophages	CC chemokine	The excessive release of cytokines and chemokines such as CCL22	[78]
Gruber C, et al.	USA		CCR4, CCR10	Review	CCL22, CCL28	MSC, T cell, macrophage, DC	T cell/DCs interaction Migration of macrophages	CC chemokine	Cytokine profiling identified elevated signatures of d mucosal immune dysregulation (IL-17A, CCL20, and CCL28).	[79]

#### 7.2.8. CCL22

It has been shown that some chemokines and cytokines, such as CCL22 as well as interleukin- and interferon-associated genes such as IFI44, IFIH1, IL10, and IFIT1, are appreciably greater in the cases with severe clinical presentations in comparison to moderate cases. Moreover, analyses of differential gene expression have shown a small set of regulatory genes that may possibly serve as robust predictors of patients’ outcomes. This could present worthwhile information on the pathophysiology of COVID-19 [80].

#### 7.2.9. CCL28

Gruber et al. also presented the profile of the secreted immune responses through high-dimensional cytokine assays that determined the multiplied signatures of chemokines such as CCL28. The release of different cytokines and chemokines such as CCL22 as well as specific interleukins, interferons, and associated genes such as interferon induced protein 44 (IFI4), interferon induced with helicase C domain (IFIH1), IL10, and IFIT1 were considerably greater in individuals with severe presentation in comparison to the mild cases. Finally, analyses of the differential gene expression showed a small set of regulatory genes that can act as the major predictors of affected persons and their final outcomes. This can offer precious insights into COVID-19 pathophysiology [80].

## 8. Chemokines and COVID-19 Vaccine Covaxin

CXC and CC sub-groups of chemokines are known to play a vital role in host immunity to viral infections. These chemokines act by inducing the activation and migration of innate and adaptive immune effectors to the site of infection. In the only study so far [81], which dealt with the chemokine responses following an entirely inactivated Covaxin COVID-19 vaccine administration, manufactured in India, it was clearly demonstrated that there was an elevated induction of CCL4, CXCL1, CXCL2, and CX3CL1chemokines, a fact that indicates the activation of innate immune cells. However, the plasma levels of CCL2 and CXCL10 following the vaccination were diminished. Nevertheless, the increased production of certain chemokines following prime-boost vaccination with Covaxin reflects the robust induction of host immunity that is of a protective nature by this inactivated vaccine. The induction of systemic cytokine and chemokine responses following COVID-19 vaccination seems to have important implications in terms of understanding the nature of the systemic immune response triggered by COVID-19 vaccines and its effect on bystander immune responses. The chemokines described in this review can be used as the surrogates of vaccine-induced innate and adaptive protective responses and these chemokines could also help in the improvement of vaccines’ efficacy and pertinence.

## 9. Systemic Signature of IP-10/CXCL10 Chemokines after BNT162b2 mRNA Vaccine

Several of the cytokines and chemokines induced by viral infection were also elevated after vaccination with mRNA vaccines, but important differences remain to be highlighted. Indeed, during inflammation, several cells including leukocytes, neutrophils, eosinophils, monocytes, and stromal cells, in response to IFN-g, can release the chemokine IP-10/CXCL10. This chemokine promotes the chemotaxis of CXCR3 cells, which are mainly activated T and B lymphocytes [82]. The secretion of IP-10/CXCL10 by IFN-g can be induced via a mechanism by which IL-15 indirectly acts on dendritic cells and macrophage/monocytes as proposed by Bergamaschi et al. [83]. Higher vaccine-induced antibody titers were associated with serum IP-10/CXCL10 levels, an innate signature linked to IP10/CXCL10 and IFN-related genes [84]. A recent study [85] demonstrated that upon vaccination with theBNT162b2 mRNA vaccine an early but transient inflammatory cytokine response takes place. Indeed, following vaccination, several cytokines including IP-10/CXCL10, IL-6, IFN-g, and CRP increased acutely at day 2 and returned to baseline levels after 8 days. Moreover, the chemokines I MIP-1a/CCL3, MIP-1b/CCL4, L-8, and IL-16 were effective up to day 8 after their release. The authors of this study supported the view that the vaccine-induced upregulation of the anti-inflammatory molecule IL-1Ra may also indicate a self-modulatory and limiting inflammatory effect of the vaccine. Importantly, these data suggest that mRNA vaccination is associated with a cytokine signature featuring CXCL10, IP-10/IL-15, and IFN-g. These authors suggested that the early cytokine/chemokine signature featuring IP-10/CXCL10, IL-15, and IFN-g, could be used to monitor the effectiveness of vaccination and as a guide to optimize the efficacy of mRNA vaccination.

## 10. Chemokines and Vaccine-Induced Immune Thrombotic Thrombocytopaenia

Chemokine (C-X-C motif) ligand 4 (CXCL4) is also known as platelet factor 4 (PF4). PF4 constitutes a component of the innate immune response of multiple cell types following infection by various pathogens. These cells include platelets, monocytes, and macrophages. Thrombocytopenia with thrombosis and especially serious cerebral venous sinus thrombosis, in the absence of external heparin administration, is indeed a rare manifestation following ChAdOx1 nCov-19 and Ad26.COV2.S vector vaccines. The main characteristics of this vaccine complication is the presence of high levels of the chemokine PF4-polyanion complexes.PF4 can quickly bind to either exogenous or endogenous heparin and constitutes a specific chemokine protein present in the a-granules of platelets. The heparins belong to endogenous glycosaminoglycans or mucopolysaccharides that are long, linear polysaccharides consisting of repeating disaccharide units (i.e., two-sugar units). Heparan sulphate and dermatan sulphate are glycosaminoglycans that when released into the bloodstream can cause severe bleeding. A recent study demonstrated that high levels of anti-PF4 antibody isotypes, endogenous glycosaminoglycans, and inflammatory biomarkers are associated with the severity of pulmonary embolisms and mortality according to a recent study [86]. The pair of PF4/heparin acts as an autoantigen and induces anti-PF4/heparin antibodies of IgG class. The triplet PF4-heparin-IgG antibody complexes can cause thrombosis via an activation of specific low-affinity IgG (FcγRIIa) receptors situated in the platelet surface. A fact that is not so well known to physicians is that platelets also contain high affinity IgE (FcεRI) and low affinity IgE (FcεRII/CD23) receptors that further facilitate thrombotic events via hypersensitivity pathways [87,88]. The extensive thrombosis increases the consumption of platelets leading to thrombocytopenia. It is worth mentioning that the classical heparin-induced thrombocytopenia has never been complicated by serious cerebral venous sinus thrombosis, as the vector vaccines have, despite constituting a highly pro-thrombotic condition. Any form of heparin such as unfractionated heparin, even for line flushes, or low molecular weight heparin e.g., enoxaparin and platelet transfusion should be avoided in COVID-19-vaccination-associated cerebral venous sinus thrombosis. Treatment with Argatroban, which is a direct thrombin inhibitor together with corticosteroids such as dexamethasone and immunoglobulin, is recommended. Therefore, specific chemokines are involved in autoimmunity with all the above described rare but serious vaccine complications.

## 11. Interplay of Cell Mechanisms in COVID-19 Involving Cytokines

Cytokines are small proteins (peptides) that are involved in autocrine, paracrine, and endocrine signaling as immunomodulating agents but cannot cross the lipid bilayer of cells to enter the cytoplasm. There are different types of cytokines associated with the severity of COVID-19 infection, which include the interleukins consisting of the largest group of cytokines, the chemokines emerging as the second largest family of cytokines, the interferons so named because they interfere with virus replication, the tumor necrosis factors, and the colony-stimulating factors [89]. Viral infection begins with the detection of viral nucleic acid by host cell pattern recognition receptors (PRRs), which signal downstream via recruited adaptor proteins, ubiquitin ligases, and kinases, culminating in transcription factors and the ultimate expression of immune genes, including IFNs, cytokines, and chemokines [90]. Furthermore, virally mediated cell death causes the release in various damage-associated molecular patterns (DAMPs) and pathogen-associated molecular patterns (PAMPs), which are believed to be recognized by cell pattern recognition receptors on alveolar macrophages and endothelial cells [91]. Currently, there is conclusive evidence that the association of a robust cytokines and especially chemokine production with a delayed or absent IFN-I and IFN-III response implies uncontrolled inflammation and constitutes a major factor for the development of severe COVID-19 [92]. Indeed, elevated levels of various interleukins such asIL-6, IL-1β, TNF-α, IL-10, and IL-8 and the chemokine MCP1 have been well documented in patients with severe COVID-19 [93]. In response to pattern recognition receptor ligands of pathogen- or damage-associated molecular patterns, the cytokine release syndrome is usually initiated by macrophages, dendritic cells, natural killer (NK) cells, and T cells [94]. The deregulated activation of macrophages plays a significant role in increasing the level of cytokines in COVID-19 infection [94]. However, it is still not confirmed whether the epithelial and endothelial cells participate in the inflammatory response to COVID-19. In a MERS-CoV infection [95], the epithelial and endothelial cells induced significant but delayed IFN and pro-inflammatory cytokine (IL-1β, IL-6 and IL-8) responses, while a large quantity of CCL3, CCL5, CCL2, and CXCL10 was produced during a SARS-CoV infection [96]. COVID-19 infection associated with inflammation may affect the vascular system and can contribute to microcirculatory lesions. An imbalance in cytokine production associated with the activation of macrophages and with the release of pro-coagulant factors, such as plasminogen activators, and the enhanced expression of plasminogen activator inhibitor-1 can increase vascular inflammation. Moreover, in patients with a severe disease stage, it promotes a prothrombotic state resulting in higher levels of IL-6 and D-dimers [97]. A condition that can act as a negative regulator of inflammation and return to equilibrium and that can further affect several pathways is autophagy. Autophagy is also named autophagocytosis, from the Ancient Greek αὐτόφαγος, autóphagos, meaning “self-devouring”, and κύτος, kýtos, meaning “hollow”, which is the natural, conserved degradation of the cell that removes unnecessary or dysfunctional components through a lysosome-dependent regulated mechanism [98]. Autophagy regulates IL-1β production by the direct destruction of inflammasomes (large intracellular multiprotein complexes that play a central role in the innate immune system, activating inflammatory responses also known as pyroptosomes) via inflammasome ubiquitination [99], removes inflammasome-activating stimuli from the cytosol [97], and denominates mitophagy (the removal of damaged mitochondria), thus preventing the release of cytosolic mtDNA and the accumulation of reactive oxygen species (ROS). The proinflammatory response might be regulated by the autophagic degradation of pivotal molecules such as MyD88, the nuclear factor kB (NF-κB), and depolarized mitochondria and ROS. Moreover, autophagy can regulate IL-1β production via the removal of ASC (apoptosis-associated speck-like protein) pyroptosome, pro-caspase 1, and pro-IL-1 [92]. Overall, autophagy is an excellent mechanism closely related to the inflammatory response. It has been suggested that its downregulation might be essential for the prevention of the pathophysiology of inflammatory diseases such as COVID-19 [92].

## 12. Different Chemokines in the Evolution of COVID-19

In symptomatic or asymptomatic COVID-19, the CCL3, CCL4, and CCL5 chemokines were detected in a similar fashion [100]. A significant increase in CXCL2, CXCL8, CXCL9, and CXCL16 levels was reported in COVID-19 positive patients with generalized inflammation [101]. Specifically, CXCL9 and CXCL16 recruited T and NK cells, CCL8 and CCL2 recruited monocytes and macrophages, and CXCL8 and CXCL2 recruited neutrophils. These cells constitute the main immune cells infiltrating the lungs of COVID-19 patients [102,103]. Symptomatic patients demonstrated higher levels of CXCL10, CCL2, and CXCL9 compared to convalescent cases [100]. According to a recent study [104], the levels of CCL4 and CCL5 were increased in all three groups of patients suffering from COVID-19, namely, mild, severe, or fatal, but these chemokines were significantly higher only in mild cases. A possible explanation for this is that CCL4 and CCL5 are likely to be associated with the recovery and resolution of inflammation possibly through the activation of cytotoxic T cells and the release of CCL5 upon antigen presentation [105,106]. Furthermore, elevated CCL5 levels remained consistently high during the one month follow up period [106]. However, some other studies have shown contradictory data regarding the CCL5 chemokine presence in severe patients as well as its close association with disease progression [107]. In another clinical investigation, COVID-19 patients requiring ICU admission exhibited higher levels of CXCL10, CCL2, CCL3, and CCL7 compared to mildly infected patients [108]. In fatal COVID-19 cases, the chemokines CXCL8, CCL2, and CCL3were significantly elevated, but they were increased similarly in mild and severe cases during the early stages of infection and remained at steady levels afterwards in the mild cases. However, these molecules were further increased during the late stages of the infection, as reported in the fatal cases [106].

Based on the above, the chemokine profile seems to constitute an important tool for the stratification and identification of patients who are at a higher risk for the development of complications in severe cases.

## 13. Limitations and Perspectives

This review regarding the COVID-19 immune signature, similar to other reviews on the same topics, identified several limitations, including differences in population demographics, a lack of negative control recruitment due to the restrictions imposed by the pandemic, variability in the laboratory assays used to evaluate chemokine levels, associated co-morbidities or co-infections, and a limited study scope to gain a holistic immune profile. Despite these limitations, there is a real consensus regarding the chemokine profile among COVID-19 patients, in which the variety of CXC and CC chemokines and their association with COVID-19 vaccination are major contributors to the immunopathology post-SARS-CoV-2 infection and remain as important targets for therapy. Based on the predictive and prognostic values of the chemokine profile, these molecules will be useful for inclusion in the routine laboratory tests of COVID-19 patients. During the COVD-19 infection stages, the analysis of chemokines and their receptors will help in identifying the possible outcome of the disease and the likelihood of the development of complications. Further studies, however, with larger groups of patients and different ethnic backgrounds are needed in order to expand our knowledge and reach a useful conclusion on the relationship between COVID-19 and chemokines, which could be beneficial in predicting the disease outcome.

## 14. Conclusions

Optimistically, advancements in our knowledge of the mechanisms and sufferer-associated elements using the resulting outcomes may be beneficial for the development of efficient preventive approaches and/or therapeutic alternatives. With regard to the cutting-edge expertise, “cytokine storm” has been shown to be a harmful and probably life-threatening condition associated with COVID-19 and its principal medical effects. Therefore, immune-mediated activities associated with responses to SARS-CoV-2 infection as well as the positions of chemokine and chemokine-receptor machinery should be widely described with the resulting aim to pick out focused treatment techniques. Although lessons can be taken from the earlier MERS and SARS epidemics, there is still room for the determination of SARS-CoV-2 behavior relative to its predecessors. Searching for challenges between viruses, current vaccines and immune defenses may assist our practical knowledge of the extraordinarily variable range of COVID-19 clinical manifestations. These manifestations seem to be different between the asymptomatic cases and the severe bilateral pneumonia as well as the several other life-threatening forms of clinical multi-organ failure.

## Data Availability

Not applicable.

## Abbreviations

AGEs	Advanced glycation end products
AIDS	Acquired immunodeficiency syndrome
ARDS	Acute respiratory distress syndrome
ARG1	Arginase 1
BALT	bronchus-associated lymphoid tissue
C1S	Complement C1s
CCL2	(C-C-motif) ligand 2
CRRT	Continuous renal replacement therapy
CXCR4	C-X-C Motif Chemokine Receptor 4
ECMO	Extra-corporeal membrane oxygenation
ELANE	Elastase, Neutrophil Expressed
GPCRs	G-protein coupled receptors
HBV	Hepatitis B virus
HIT	Heparin-induced thrombocytopenia
HIV	Human immunodeficiency virus
INF	Interferon
IFIH1	Interferon Induced with Helicase C Domain 1
IFI44	Interferon Induced Protein 44
IL-6|	Interleukin 6
LPS	Lipo-polysaccharide
MERS-CoV	Middle East respiratory syndrome coronavirus
MIP	Macrophage Inflammatory Proteins
MIS-C	Multisystem Inflammatory Syndrome in children
NAPS2	Not Another PDF Scanner 2
PF4	Platelet factor 4
PTGS2	Prostaglandin-Endoperoxide Synthase 2
RANTES	Regulated on Activation, Normal T Cell Expressed and Secreted
ROS	Reactive Oxygen Species
SARS-CoV-2	Severe acute respiratory syndrome coronavirus-2
SLE	Systemic Lupus Erythematosus
TNFRSF1A	TNF Receptor Superfamily Member 1A
TNF	tumor necrosis factor
TRM	tissue-resident reminiscence T
TYMP	Thymidine Phosphorylase
XIAP	X-linked inhibitor of apoptosis protein
